# Respiratory Adenovirus Quantification with a Droplet Digital Polymerase Chain Reaction (ddPCR) Assay

**DOI:** 10.1128/spectrum.00269-23

**Published:** 2023-04-18

**Authors:** Omar Abdullah, Amary Fall, Michael Forman, Craig Howard, Eili Klein, Heba H. Mostafa

**Affiliations:** a Johns Hopkins School of Medicine, Department of Pathology, Division of Medical Microbiology, Baltimore, Maryland, USA; b Department of Emergency Medicine, Johns Hopkins School of Medicine, Baltimore, Maryland, USA; c Center for Disease Dynamics, Economics, and Policy, Washington, DC, USA; University of Utah and ARUP Laboratories

**Keywords:** adenovirus, droplet digital PCR, ddPCR

## Abstract

Human adenoviruses (HAdVs) are double-stranded DNA viruses that can cause a wide spectrum of disease, including respiratory infections. Little is known about the value of respiratory HAdV quantification and its correlation with disease severity. In this study, we developed a quantitative HAdV droplet digital PCR (ddPCR) assay to study the association between viral loads, circulating types, and clinical outcomes. Remnant respiratory specimens positive for HAdV after the standard of care testing were collected from December 2020 to April 2022. A total of 129 samples were tested by a ddPCR method. Typing was performed using Nanopore sequencing of the hexon gene hypervariable region. Clinical chart reviews were performed to correlate the viral loads with the disease severity. The ddPCR assay showed an analytical sensitivity and a lower limit of quantification below 100 copies/mL. Of 129 positive clinical samples, 100 were quantified by ddPCR, 7 were too concentrated to be quantified, and 22 were negative. Of the 22 false negatives, only 3 were successfully typed; however, 99 of the 107 positive samples had a characterized genotype. The main HAdV types identified in this cohort were C1 (49.5%) followed by C2 (34.3%). No significant difference in HAdV loads was noted between patients who were admitted, those who required supplemental oxygen, and outpatients or between different HAdV types. HAdV ddPCR is a reliable absolute quantification approach for HAdV from respiratory samples. HAdV loads at initial presentation does not appear to differ between patients who require hospitalization versus outpatients.

**IMPORTANCE** Measuring viral load using droplet digital PCR (ddPCR) is an absolute quantification approach that can facilitate comparability between different laboratories. This approach could prove valuable in studies that focus on the clinical utility of quantification. In this study, we evaluate a human adenovirus (HAdV) ddPCR assay and study the relationship between viral loads and outcomes after HAdV respiratory infections.

## INTRODUCTION

Adenoviruses belong to *Adenoviridae* family that characterize with double-stranded nonenveloped virus particles and can cause a large spectrum of disease (1, 2). Human adenoviruses (HAdVs) belong to seven species (A through G) within the Mastadenovirus genus. Infections by HAdVs are ubiquitous, and up to 24% of respiratory infections and 15% of cases of diarrhea in children are caused by HAdVs (3). Infections in immunocompromised patients can be disseminated and are associated with high mortality (4).

HAdV respiratory infections are more associated with the B, C, and E species. Types 1, 2, 5, and 6 are associated with endemic infections; however, types 4, 7, 14, and 21 are associated with outbreaks. Type 21 can cause severe lower respiratory disease, and types 4 and 7 were historically associated with severe respiratory disease in military recruits. Gastroenteritis cases are primarily associated with types 40 and 41; however, respiratory infections with other types can be associated with gastroenteritis (1).

The molecular detection of HAdV in respiratory specimens has become the gold standard for diagnosing HAdV respiratory infections (5). Commercially available and laboratory-developed tests for respiratory HAdV infections are widely used clinically with different chemistries but are largely qualitative (6–9). Laboratory-developed tests have been mainly real-time PCR that can be validated quantitatively with appropriate external standards. The major utility of quantitative HAdV assays has been monitoring HAdV DNAemia for cases of disseminated infections or in immunocompromised patients (10–12). Quantification of HAdV from respiratory samples by real-time PCR was investigated to assess the correlation of respiratory disease severity and viral loads (13–15). In this study, we evaluated the performance of a droplet digital PCR HAdV assay and examined its utility for correlating HAdV loads in upper respiratory specimens with disease and viral types.

## RESULTS

### Analytical characterization of the adenovirus ddPCR assay.

**FIG 1:**
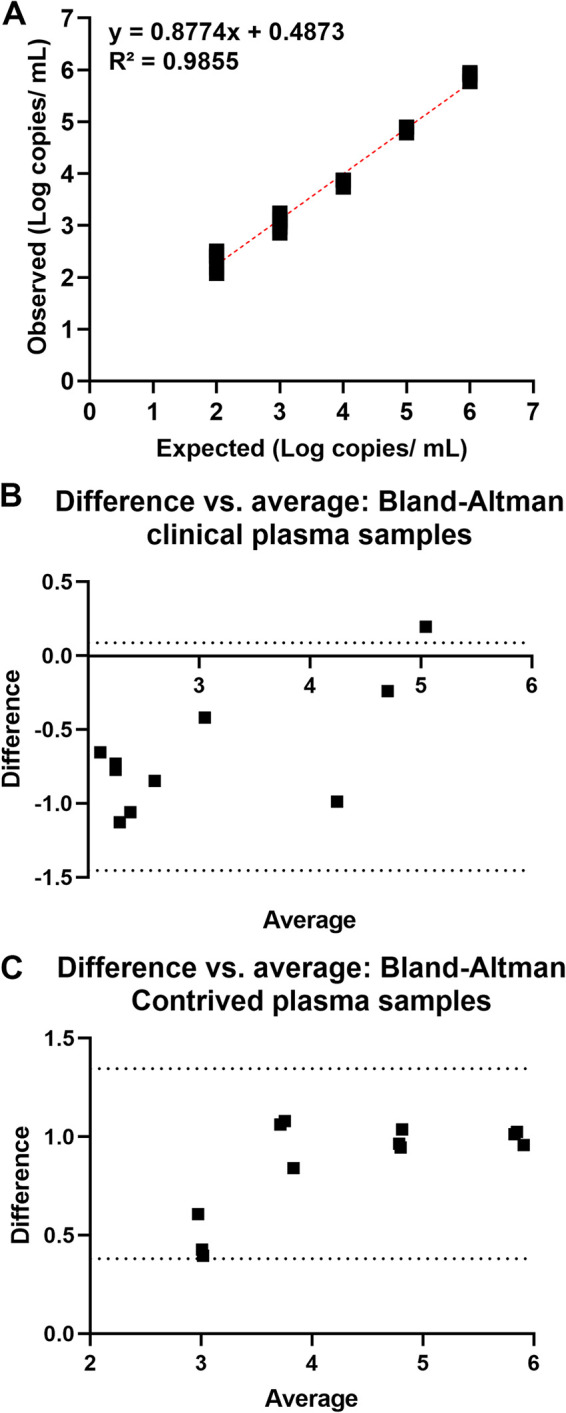
Analytical performance of the human adenovirus (HAdV) droplet digital PCR (ddPCR) assay. (A) Standard curve using serially diluted adenovirus stock in Universal Viral Transport media. (B) Quantitative correlation of the HAdV ddPCR assay with the clinical standard of care quantitative HAdV real-time PCR test for remnant, previously positive plasma samples. (C) Quantitative correlation of the HAdV ddPCR assay with the clinical standard of care quantitative HAdV real-time PCR test for contrived plasma samples.

A serially diluted adenovirus quantified stock (8) was run by the HAdV droplet digital PCR (ddPCR) assay (dilutions were made in universal viral transport media). All replicates were detectable to a concentration of 100 copies/mL (Table 1). The observed concentration showed an excellent quantitative correlation with the expected concentration (Fig. 1A) with an average difference of 0.05 log copies/mL. The assay’s precision was high for tested replicates with a maximum standard deviation of 0.16 log copies/mL, which was notable at the lowest concentration of 200 copies/mL (Table 1).

**TABLE 1:** HAdV ddPCR analytical measurement range and reproducibility^*a*^

Expected (log copies/mL)	Replicates (*N*)	Observed (mean log copies/mL)	SD (log copies/mL)
6	3	5.876997	0.090737
5	3	4.860101	0.061567
4	3	3.829334	0.076821
3	6	3.058738	0.135513
2	6	2.33155	0.165624

a ddPCR, droplet digital PCR; HAdV, human adenovirus.

A cohort of plasma specimens was used to assess the quantitative correlation of the HAdV ddPCR with the clinical quantitative assay used for the standard of care viral load HAdV testing. A total of 20 plasma specimens were tested, of which 9 were negative by the HAdV ddPCR assay. The average viral load of the false-negative plasma samples was 1.75 log copies/mL (Table 2). A mean bias of −0.68 log copies/mL (standard deviation [SD], 0.39; 95% limits of agreement: lower, −1.454 and upper, 0.087) was observed for this cohort of samples (Fig. 1B). To evaluate the quantitative correlation of the two assays side by side using the same extraction method, a contrived panel was made by spiking HAdV negative plasma samples with the adenovirus stock (Virapur [8]). Serial dilutions were made in plasma matrix and tested in replicates side by side with both the standard of care test and the HAdV ddPCR test. Four samples with a concentration of 100 copies/mL or less were not detected by the HAdV ddPCR but were detected by the standard of care method (Table 3). A mean difference of 0.57 log copies/mL was observed with a mean bias of 0.86 log copies/mL (SD, 0.25; 95% limits of agreement: lower, 0.38 and upper, 1.345) (Fig. 1C).

**TABLE 2:** Agreement of the HAdV ddPCR with the standard of care quantitative HAdV assay for positive plasma specimens^*a*^

Standard of care HAdV load testing (log copies/mL)	HAdV ddPCR (log copies/mL)
1.53	2.41
1.78	2.43
1.88	2.61
1.86	2.63
1.72	2.85
1.85	2.91
2.18	3.02
2.84	3.26
3.75	4.74
4.58	4.82
5.14	4.94
1.88	ND
1.25	ND
1.96	ND
1.75	ND
1.95	ND
1.35	ND
2.08	ND
2.06	ND
1.46	ND

a ND, not detected.

**TABLE 3:** Agreement of the HAdV ddPCR with the standard of care quantitative HAdV assay for contrived plasma specimens^*a*^

Expected (log copies/mL)	Standard of care HAdV load testing (log copies/mL)	HAdV ddPCR (log copies/mL)
6	6.33	5.32
6	6.36	5.34
6	6.39	5.43
5	5.27	4.30
5	5.33	4.29
5	5.27	4.33
4	4.29	3.21
4	4.25	3.41
4	4.24	3.18
3	3.22	2.79
3	3.28	2.67
3	3.22	2.82
2	2.21	ND
2	1.96	2.70
2	2.08	ND
1	<1.18	ND
1	1.18	2.84
1	1.59	ND

a ND, not detected.

### HAdV quantification by ddPCR from respiratory samples, December 2020 to April 2022.

Between December 2020 and April 2022, a total of 173 respiratory samples were positive for HAdV with an average positivity rate of 1.6%. A total of 129 positive samples (by the standard of care ePlex panels) were collected and tested by the HAdV ddPCR assay; 22 samples were negative, 7 were too concentrated to be quantified, and 100 were quantified by ddPCR (Table S1). An internal control human gene (RNAseP) was quantified using ddPCR to control for sample sufficiency when comparing viral loads between different groups. For the whole cohort (129), an average of 5.3 log copies/mL RNAseP was noted with SD of 0.6 log copies/mL (Table S1). Genotyping was performed for the whole cohort of 129 respiratory samples, which showed a predominance of genotypes C1 (49.5%) followed by C2 (34.3%) (Table S1). Of the 22 false negatives, only 3 were successfully typed; however, 99 of the 107 positive samples had a characterized genotype (Table S1).

### Viral loads and associations with disease and genotypes.

**FIG 2:**
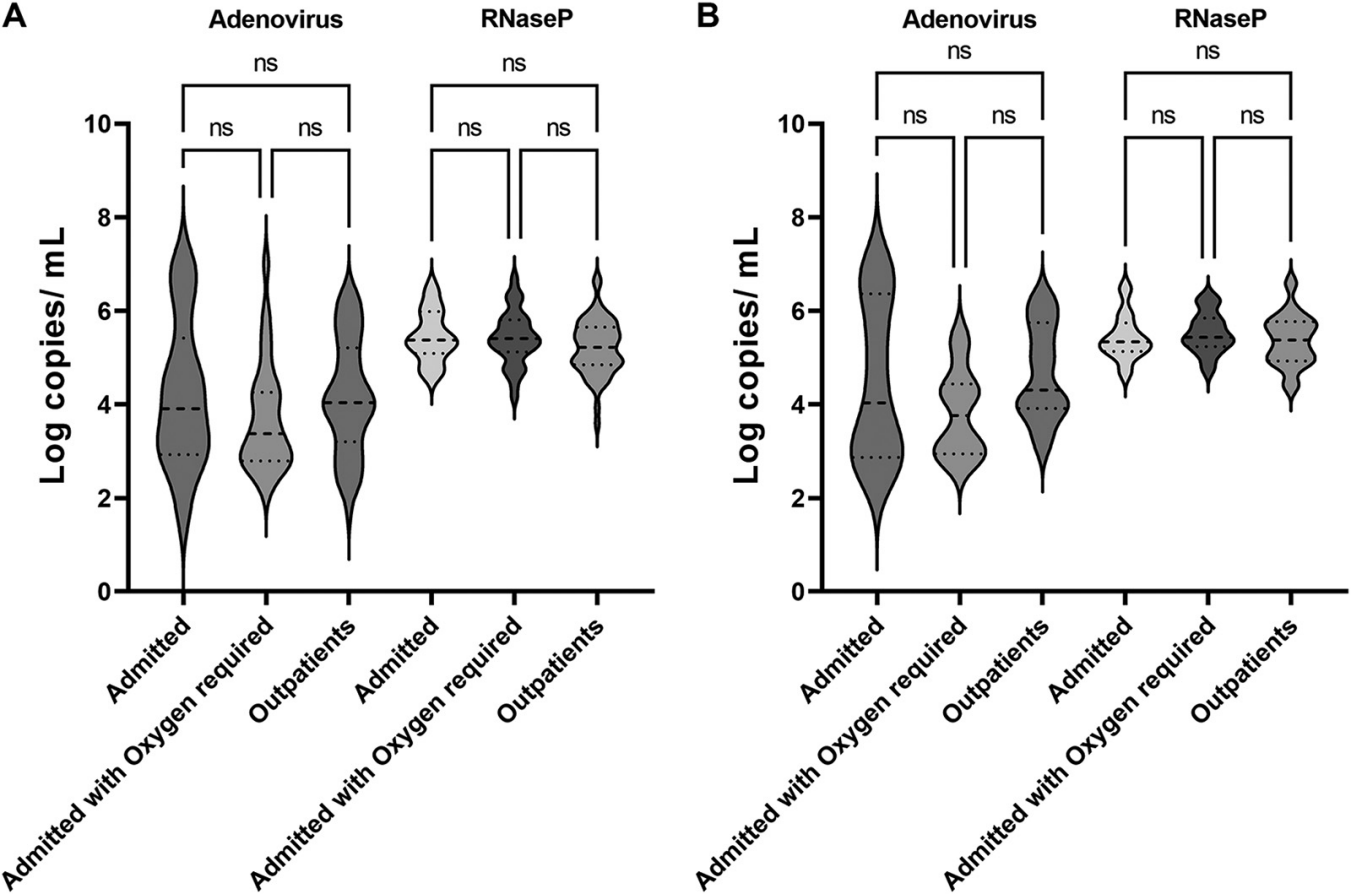
Association of HAdV loads in respiratory samples with clinical disease outcomes in the whole cohort with complete clinical data (A) and the cohort with signs of respiratory infections at encounter (B). ns, not statistically significant.

**FIG 3:**
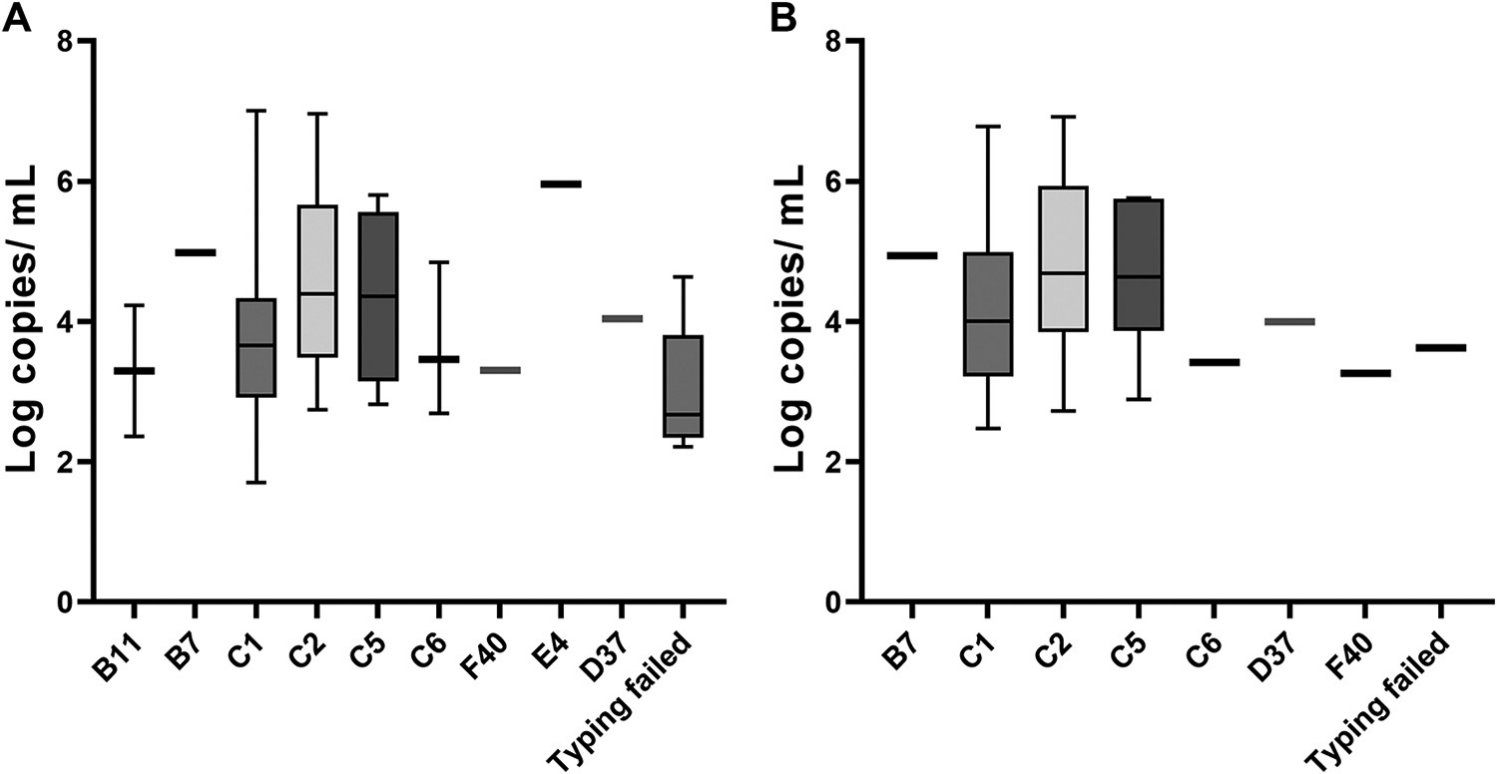
Association of HAdV loads in respiratory samples with genotypes in the whole cohort with complete clinical data (A) and the cohort with signs of respiratory infections at encounter (B).

To correlate the viral loads with the disease outcomes, clinical chart reviews were performed, and complete clinical data including symptoms at presentation were available for 68 patients. The main presenting sign was fever in 54.4%, followed by gastrointestinal tract symptoms that included abdominal pain, vomiting, and diarrhea in 19.1% of patients (Table 4). The median age of the whole cohort was 2 years with 44% admitted, 15% requiring intensive care unit (ICU) level care, and 29% requiring supplemental oxygen. The main underlying condition observed in the cohort was immunosuppression in 34% of patients. Viral loads were not different in outpatients versus admitted or admitted with oxygen required (Fig. 2A). When the analysis was limited to the cohort of patients with signs of respiratory infections at encounter (N = 56; Table 4), viral loads were not different in outpatients versus admitted or admitted with oxygen required (Fig. 2B). Viral loads were not different between different HAdV types when the whole cohort was used (Fig. 3A) or when the analysis was limited to patients with signs of respiratory infections at encounter (Fig. 3B).

**TABLE 4:** Clinical and metadata of patients with quantified HAdV in respiratory samples by the HAdV ddPCR assay^*a*^

Characteristics	No. of patients	Result
Total (with quantified HAdV with ddPCR)	100	2 yr (18.6)^*b*^
With known clinical signs	68	1.5 yr (13.3)^*b*^
With signs of respiratory infection at encounter	56	1 yr (3.8)^*b*^
Clinical signs		
Fever	40	58.82^*c*^
GIT (abdominal pain, diarrhea, vomiting, dehydration)	13	19.12^*c*^
Difficulty breathing, chest pain	10	14.71^*c*^
Neurological (altered mental status, seizures)	4	5.88^*c*^
Other signs (abnormal lab work)	1	1.47^*c*^
Outcome		
Admitted	44	44^*d*^
ICU	15	15^*d*^
Supplemental oxygen	29	29^*d*^
Outcome		
Admitted	22	39.3^*e*^
ICU	8	14.3^*e*^
Supplemental oxygen	12	21.4^*e*^
Comorbidities (total included)		
Hypertension	25	25^*f*^
Pregnancy	1	1^*f*^
Lung disease	31	31^*f*^
Kidney disease	21	21^*f*^
Immunosuppression	34	34^*f*^
Diabetes	11	11^*f*^
Heart failure	5	5^*f*^
Atrial fibrillation	3	3^*f*^
Smoker	5	5^*f*^
Cerebrovascular disease	7	7^*f*^
Cancer	25	25^*f*^
Coronary artery disease	7	7^*f*^

a GIT, gastrointestinal tract; ICU, intensive care unit.

b The values shown are the patients’ ages (standard deviation).

c The values shown are percentages of 68 total with known clinical signs.

d The values shown are percentages of 100 total with HAdV quantified by ddPCR.

e The values shown are percentages of 56 total with signs of respiratory infection at encounter.

f The values shown are percentages of 100 total with HAdV quantified by ddPCR.

## DISCUSSION

In this study, we evaluated the performance of a ddPCR assay for HAdV and assessed the association between HAdV loads in respiratory samples with disease outcomes. The ddPCR assay had an excellent correlation with the ddPCR assay used by Virapur (now Microbiologics) to quantify their commercial adenovirus stock (as evident by the standard curve analysis) and excellent reproducibility. The assay showed lower clinical sensitivity than the standard of care tests (respiratory and plasma) for samples with loads at or less than 100 copies/mL. The quantitative correlation with the standard of care HAdV quantitative test (for plasma samples) showed bias that was different when remnant clinical samples were used for comparison versus a contrived plasma panel. When remnant plasma samples were used, the majority of the samples had quantities less than 100 copies/mL, a concentration range at which the HAdV ddPCR assay showed an average increase in quantitation of 0.87 log copies/mL. Notably, the clinical assay used a different extraction method and different extraction and elution volumes. On the other hand, using a contrived panel in plasma matrix, an average reduction of 0.57 log copies/mL was notable, which was close to 1 log for samples with quantities above 10,000 copies/mL. The clinical archived samples had a median log value of 1.88 copies/mL and an average of 2.64 log copies/mL, in contrast to a median of 4.78 and an average of 4.78 log copies/mL for the contrived samples. This difference in viral load range can lead to a difference in the calculated mean bias. Overall, our data show excellent linearity of the ddPCR assay, even if its absolute quantification shows a biased correlation with the standard of care quantitative real-time PCR. Our data also emphasize that standardizing the extraction methods contributes to the commutability of quantification across different assays.

The clinical utility of respiratory virus quantification is debatable. During the COVID-19 pandemic, relative quantification of severe acute respiratory syndrome coronavirus 2 (SARS-CoV-2) in respiratory samples using cycle threshold values was used by providers in certain clinical situations to guide treatment or infection prevention (16–18). The lack of standardized approaches for quantification of respiratory viruses along with other variables that include the specimen types used for diagnosis challenges our understanding of the clinical significance of viral loads and their correlation with disease severity (11, 19–24). In our study, no significant differences in HAdV loads were noted between patients who were admitted, those who required supplemental oxygen, or outpatients. No significant differences were observed between patients infected with different genotypes either. A recent study showed that high HAdV cycle threshold values (lower relative viral loads) correlate with the disease severity in admitted pediatrics patients (13). In this study, the authors discussed that an important variable is likely the stage of the disease when patients were hospitalized. Our study compared viral loads between patient groups at their initial encounter, which was primarily within the first week of the onset of symptoms. HAdV load peaks during the first week of symptoms (15) and hence a proper assessment requires prospective daily collection from outpatients and inpatients and comparing viral loads between groups in relation to the onset of symptoms, looking particularly at the duration of viral shedding in inpatients versus outpatients and the viral load difference at later time points after infection.

The limitations of our study include the exclusion of multiple patients’ charts due to incomplete clinical information. In addition, a limited population of patients receive testing for HAdV, which likely include those who present to the emergency departments, pediatrics patients, and those who require hospitalization, which limits the data of individuals with asymptomatic or mild disease. Moreover, prolonged shedding of the virus that can extend for more than 60 days (15) can challenge the interpretation of molecular results.

In conclusion, HAdV ddPCR is an accurate absolute quantification approach that can be commutable between different laboratories and hence is appropriate for clinical studies that aim at understanding the clinical utility of quantification. Viral loads in respiratory specimens at initial presentation do not appear to correlate with the disease outcomes.

## MATERIALS AND METHODS

### Ethics and study samples.

The research was conducted with a waiver of consent under Johns Hopkins Institutional Review Board (IRB) protocol IRB00247284. Respiratory samples were remnant specimens that were positive for HAdV by the standard of care ePlex respiratory pathogen panels (8). A total of 129 respiratory samples were collected from December 2020 to April 2022 for the study. Samples included 123 nasopharyngeal swabs, 5 bronchoalveolar lavage specimens (BAL), and 1 throat swab; of these, only 100 were positive with the HAdV ddPCR (95 nasopharyngeal swabs, 1 throat swab, and 4 BAL specimens). Notably, 30 HAdV negative respiratory samples were tested by the HAdV ddPCR assay to assess the assay’s clinical specificity and were all negative. Plasma samples were used for evaluating the analytical performance of the HAdV ddPCR assay to compare the HAdV ddPCR quantification to the clinically validated quantitative HAdV assay used for standard of care (quantification if performed clinically only from plasma). For that, a total of 20 plasma samples positives for HAdV in addition to 24 HAdV negative plasma samples that were used to make a contrived panel were used for the comparison. An adenovirus (quantified at 1.0 E8 copies/mL by ddPCR at Virapur [now Microbiologics] [8]) was used for the analytical evaluation of the HAdV ddPCR assay and for making the contrived panel.

### Standard of care quantitative HAdV assay.

The quantitative HAdV standard of care testing is used for quantifying HAdV from plasma samples only. The assay is a real-time PCR that uses the RealStar Adeno PCR kit 1.0 (Altona Diagnostics), eMag extraction (bioMérieux; 500 μL extracted volume and 50 μL elution volume), and HAdV standards from Exact Diagnostics (200 to 2,000,000 copies/mL). The PCR volume consists of a final volume of 30 μL (5 μL master A reagent, 15 μL master B reagent, and 10 μL extracted sample). Thermocycling conditions include a single cycle of denaturation at 95°C for 10 min followed by 45 cycles of 95°C for 15 s and 58°C for 1 min.

### HAdV ddPCR.

Samples were extracted via the Chemagic viral RNA/DNA kit following the manufacturer’s instructions (300 μL extracted volume and 60 μL elution volume). The ddPCR was carried out using the primers and probe set AQ1-F (5′-GCCACGGTGGGGTTTCTAAACTT), AQ2-R (5′-GCCCCAGTGGTCTTACATGCACATC), and AP Probe (5′-Fam-TGCACCAGACCCGGGCTCAGGTACTCCGA) previously described (25). The assays were performed according to the manufacturer’s instructions in a final volume of 22 μL consisting of 5.5 μL Supermix, 2.2 μL reverse transcriptase, 1.1 μL 300 mM dithiothreitol, 0.9 μL each primer, 0.45 μL of probe, 5.95 μL of RNase/DNase-free water, and 5 μL of extracted DNA. The droplets were generated by the QX200 automated droplet generator. After droplet generation was completed, PCR amplification was performed as follows: 50°C for 60 min, 95°C for 10 min, and 40 cycles of 94°C for 30 s and 55°C for 60 s, and then a final step of 98°C for 10 min and 4°C for 30 min (protocol used for quantifying SARS-CoV-2 yielded best results [26]). The amplified droplet plate was read using the QX200 droplet reader and analyzed with the QuantaSoft Analysis Pro 1.0.596.0525 (Bio-Rad).

### Adenovirus hexon amplification and DNA sequencing.

The hexon gene sequences of the samples screened positive for adenovirus were obtained by nested PCR amplification with the Adv Hex F1 (5′-TICTTTGACATICGIGGIGTICTIGA-3′), Adv Hex F2 (5′-GGYCCYAGYTTYAARCCCTAYTC-3′), Adv Hex R1 (5′-CTGTCIACIGCCTGRTTCCACA-3′), and Adv Hex R2 (5′-GGTTCTGTCICCCAGAGARTCIAGCA-3′) as described previously (27). Briefly, the first PCR was carried out using *Taq* DNA polymerase (Roche Diagnostics) in a 50-μL reaction volume containing 5 μL of DNA, 5 μL of 10× buffer, 4 μL of dNTPs, 1 μL of each primer (diluted at 10 μM) forward Adv Hex F1 and the reverse Adv Hex R1, 0.25 μL enzyme, and 33.75 μL RNase-free H_2_O. The amplification conditions were as follows: 94°C for 2 min, followed by 35 cycles of 94°C for 1 min, 45°C for 1 min, and 72°C for 2 min, with a final extension of 72°C for 5 min. Then 1 μL of the first PCR was added to a second PCR master mix for nested amplification using the same conditions. The PCR products were prepared for sequencing using the Native barcoding genomic DNA kit (EXP-NBD196) and the NEBNext ARTIC library prep kit according to the manufacturer’s instructions and sequenced using a R9.4.1 flowcells (Oxford Nanopore Technologies) on a GridION (Oxford Nanopore Technologies).

### Phylogenetic analysis of adenovirus sequences.

The alignment of the sequences obtained after sequencing was performed using Mafft (version 7.450). Maximum-likelihood trees were constructed using IQ-TREE2 (version 2.0.6) with 1,000 bootstrap replicates and visualized with FigTree version 1.4.4 (Fig. S1). The ModelFinder, implemented in IQ-TREE2, was used to select the best fitted nucleotide substitution model.
