# Supplementary material for: Respiratory Adenovirus Quantification with a Droplet Digital Polymerase Chain Reaction (ddPCR) Assay
Source: Microbiol Spectr. 2023 Apr 18;11(3):e00269-23. doi: 10.1128/spectrum.00269-23 (PMC10269445; doi:10.1128/spectrum.00269-23)
Supplement: Supplemental file 1 — Supplemental material. Download spectrum.00269-23-s0001.pdf, PDF file, 0.5 MB [file spectrum.00269-23-s0001.pdf]

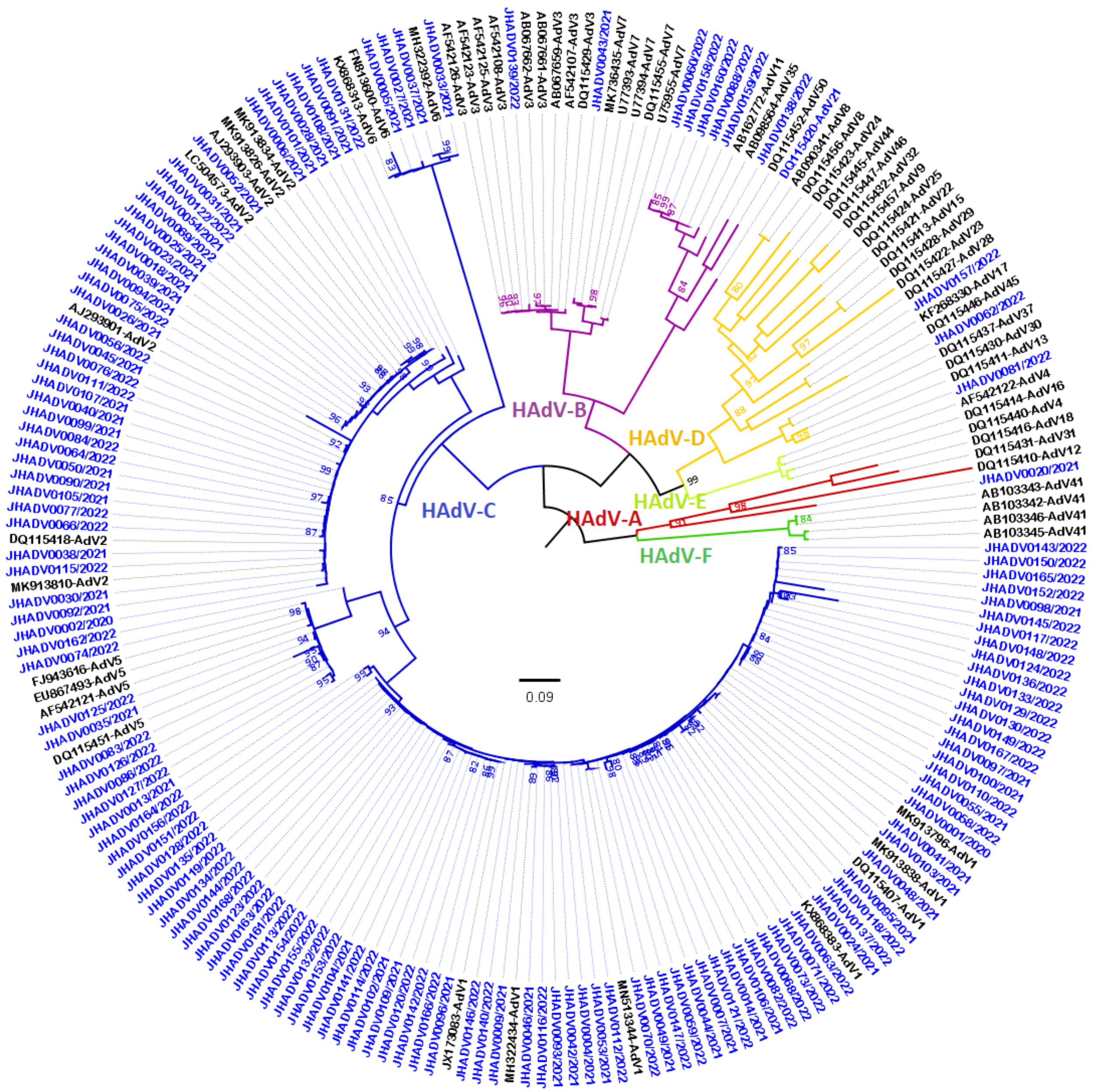

Figure S1. Phylogenetic tree of the nucleotide sequences of the hypervariable region of the adenovirus hexon gene for samples collected from December 2020 to October 2021(blue). The tree was generated with a maximum-likelihood method using Iqtree. We performed 1,000 bootstrap replications to determine the consensus tree; support for nodes present in >70% of the trees are annotated.

ADVID	Type	Quantification (copies/uL)	copies/ mL	log copies/ mL	Quantification (Internal Control, RNAse P), copies/uL	RNAse P copies/ mL	RNAse P log copies/ mL
ADV001	C1	N/A		0	272	239360	5.379051576
ADV003		N/A		0	496	436480	5.639964349
ADV004	C1	1.05	924	2.965671971	32.3	28424	4.453685194
ADV005	C6	78.6	69168	4.839905218	790	695200	5.842109763
ADV006	C2	2114	1860320	6.269587655	266	234080	5.369364309
ADV007	C1	31.2	27456	4.438637266	73.9	65032	4.813127111
ADV008		0.51	448.8	2.652052848	206	181280	5.258349893
ADV009	C1	too concentrated		0	38.7	34056	4.532193637
ADV010		N/A		0	204	179520	5.25411284
ADV011		N/A		0	288	253440	5.40387516
ADV013	C5	19.4	17072	4.232284402	376	330880	5.519670517
ADV014	C1	12.3	10824	4.034387784	730	642400	5.807805532
ADV015		N/A		0	25.8	22704	4.356102378
ADV016		N/A		0	766	674080	5.828711442
ADV017		N/A		0	19.2	16896	4.227783901
ADV018	C2	0.63	554.4	2.743823222	224	197120	5.29473069
ADV019		0.242	212.96	2.328298038	42.6	37488	4.573892271
ADV020	F40	2.28	2006.4	3.302417519	1101	968880	5.986269991
ADV021		5.27	4637.6	3.666293287	342	300960	5.478508778
ADV022		N/A		0	182	160160	5.20455406
ADV023	C2	2.82	2481.6	3.39473178	94.2	82896	4.918533575
ADV024	C1	1.15	1012	3.005180513	266	234080	5.369364309
ADV025	C2	379	333520	5.523121882	143	125840	5.09981871
ADV026	C2	N/A		0	189	166320	5.220944476
ADV027	C6	3.27	2877.6	3.459030425	440	387200	5.587935349
ADV028	C2	1612	1418560	6.15184771	1561	1373680	6.137885575
ADV029		N/A		0	9.47	8333.6	3.920832651
ADV030	C2	713	627440	5.797572202	539	474320	5.676071437
ADV031	C2	10.4	9152	3.961516011	434	381920	5.581972402
ADV032		N/A		0	911	801680	5.904001049
ADV033	C6	0.553	486.64	2.687207803	208	183040	5.262546007
ADV034		N/A		0	305	268400	5.428782511
ADV035	C5	7.17	6309.6	3.800001828	434	381920	5.581972402
ADV036		N/A		0	645	567600	5.754042387
ADV037	C6	too concentrated		0	292	256960	5.409865524
ADV038	C2	375	330000	5.51851394	330	290400	5.462996612
ADV039	C2	1.08	950.4	2.977906428	957	842160	5.92539461

ADV040	C2	12.4	10912	4.037904357	265	233200	5.367728546
ADV041	C1	33.7	29656	4.472112573	63.1	55528	4.744512031
ADV042	C1	7523	6620240	6.820873734	3674	3233120	6.509621824
ADV043	B7	108	95040	4.977906428	273	240240	5.380645319
ADV044	C1	12.7	11176	4.048286393	91.2	80256	4.90447751
ADV045	C2	10367	9122960	6.960135771	182	160160	5.20455406
ADV046	C1	too concentrated		0	282	248160	5.39473178
ADV047		8.01	7048.8	3.848115188	102	89760	4.953082844
ADV048	C1	9.46	8324.8	3.920373809	765	673200	5.828144107
ADV049	C1	212	186560	5.270818533	364	320320	5.505584056
ADV050	C2	2.33	2050.4	3.311838593	773	680240	5.832662166
ADV051		N/A		0	795	699600	5.844849801
ADV052	C2	9.24	8131.2	3.910154643	2082	1832160	6.262963397
ADV053	C1	477	419760	5.623001051	323	284240	5.453685194
ADV054	C2	18.9	16632	4.220944476	904	795520	5.900651103
ADV055	C1	2.7	2376	3.375846436	586	515680	5.712380288
ADV056	C2	957	842160	5.92539461	109	95920	4.98190917
ADV057		0.282	248.16	2.39473178	105	92400	4.965671971
ADV058	C1	21.4	18832	4.274896445	689	606320	5.782701894
ADV059	C1	too concentrated		0	1213	1067440	6.028343473
ADV060	B11	19.2	16896	4.227783901	16.9	14872	4.172369377
ADV061		0.186	163.68	2.213995616	158	139040	5.143139759
ADV062	D37	12.3	10824	4.034387784	99.3	87384	4.941431921
ADV063	C1	0.508	447.04	2.650346384	86.1	75768	4.879485824
ADV064	C2	4.33	3810.4	3.580970569	2515	2213200	6.345020662
ADV065		N/A		0	885	778800	5.891425943
ADV066	C2	91.4	80432	4.905428868	123	108240	5.034387784
ADV067		N/A		0	187	164560	5.216324279
ADV068	C1	12.6	11088	4.044853217	4771	4198480	6.623092089
ADV069	C2	39.6	34848	4.542177858	782	688160	5.837689425
ADV070	C1	13.7	12056	4.081203239	147	129360	5.111800007
ADV071	C1	2.95	2596	3.414304688	66.4	58432	4.766650752
ADV072		N/A		0	883	777040	5.890443376
ADV073	C1	0.654	575.52	2.76006042	5418	4767840	6.678321673
ADV074	C5	too concentrated		0	258	227040	5.356102378
ADV075	C2	0.822	723.36	2.85935449	141	124080	5.093701785
ADV076	C2	0.989	870.32	2.939678964	240	211200	5.324693914
ADV077	C2	194	170720	5.232284402	73.1	64328	4.808400049
ADV078		N/A		0	51.6	45408	4.657132374
ADV079		N/A		0	348	306240	5.486061916
ADV080		N/A		0	6.02	5297.6	3.724079163

ADV081	E4	1025	902000	5.955206538	65.9	57992	4.763368087
ADV082	C1	2.86	2516.8	3.400848705	56.1	49368	4.693445533
ADV083	C5	0.75	660	2.819543936	489	430320	5.633791531
ADV084	C2	28	24640	4.391640703	93.9	82632	4.917148264
ADV085		48.8	42944	4.632902494	1133	997040	5.998712582
ADV086	C5	34.7	30536	4.484812147	596	524480	5.719728932
ADV087		0.565	497.2	2.69653112	159	139920	5.145879796
ADV088	B11	0.261	229.68	2.361123179	336	295680	5.47082195
ADV089		0	0	#NUM!	241	212080	5.326499715
ADV090	C2	2708	2383040	6.377131332	1207	1062160	6.026189942
ADV091	C2	38.9	34232	4.534432273	353	310640	5.492257378
ADV092	C2	1.42	1249.6	3.096771017	542	476960	5.678481959
ADV093	C1	11512	10130560	7.005633453	316	278080	5.444169755
ADV094	C2	5.74	5051.2	3.703394565	28.7	25256	4.402364569
ADV095	C1	279	245520	5.390086875	750	660000	5.819543936
ADV096	C1	22.2	19536	4.290835647	98.2	86416	4.93659416
ADV097	C1	9.26	8148.8	3.911093659	53.4	46992	4.672023929
ADV098	C1	0.0596	52.448	1.719728932	45.5	40040	4.602494069
ADV099	C2	2233	1965040	6.293371395	175	154000	5.187520721
ADV100	C1	7.6	6688	3.825296264	33.6	29568	4.47082195
ADV101	C2	too concentrated		0	407	358160	5.554077081
ADV102	C1	4.74	4171.2	3.620261014	30.9	27192	4.434441152
ADV103	C1	366	322080	5.507963758	71.8	63184	4.800607116
ADV104	C1	2.05	1804	3.256236533	270	237600	5.375846436
ADV105	C2	1509	1327920	6.123171912	40	35200	4.546542663
ADV106	C1	122	107360	5.030842503	599	527120	5.721909495
ADV107	C2	300	264000	5.421603927	282	248160	5.39473178
ADV108	C2	40.3	35464	4.549787718	2191	1928080	6.28512505
ADV109	C1	0.233	205.04	2.311838593	4.37	3845.6	3.584964109
ADV110	C1	0.566	498.08	2.697299103	10	8800	3.944482672
ADV111	C2	23.3	20504	4.311838593	76.4	67232	4.827576031
ADV112	C1	2355	2072400	6.316473584	864	760320	5.880996415
ADV113	C1	1	880	2.944482672	55.3	48664	4.687207803
ADV114	C1	20.1	17688	4.24767873	2028	1784640	6.251550623
ADV115	C2	0.669	588.72	2.76990879	292	256960	5.409865524
ADV116	C1	too concentrated		0	399	351120	5.545455568
ADV117	C1	0.0581	51.128	1.708658805	40.4	35552	4.550864037
ADV118	C1	5.59	4919.2	3.69189448	269	236720	5.374234952
ADV119	C1	0.372	327.36	2.515025612	61.9	54472	4.736173321
ADV120	C1	0.237	208.56	2.319231018	309	271920	5.434441152
ADV121	C1	2.05	1804	3.256236533	310	272800	5.435844366

ADV122	C2	10.1	8888	3.948804046	630	554400	5.743823222
ADV123	C1	0	0	#NUM!	8.93	7858.4	3.895334131
ADV124	C1	0.22	193.6	2.286905353	252	221760	5.345883213
ADV125	C5	0.962	846.56	2.927657744	191	168080	5.225516039
ADV126	C5	81.9	72072	4.857766574	4968	4371840	6.640664259
ADV127	C5	718	631840	5.800607116	158	139040	5.143139759
ADV128	C1	0.798	702.24	2.846485564	78	68640	4.836577275
ADV161	C1	1.57	1381.6	3.140382325	203	178640	5.25197871
ADV162	C5	702	617760	5.790819784	160	140800	5.148602655
ADV163	C1	0.987	868.56	2.938799825	200	176000	5.245512668

Table S1. Samples used for the study.
